# Assessing of the glymphatic system using DTI‐ALPS MRI in patients with neurodegenerative diseases

**DOI:** 10.1002/alz.086358

**Published:** 2025-01-09

**Authors:** Alina Liaskovik, Ekaterina Fedotova, Rodion Konovalov, Marina V. Krotenkova, Yuliya Shpilyukova, Kseniya Nevzorova, Lusine Brsikyan

**Affiliations:** ^1^ Research Center of Neurology, Moscow Russian Federation

## Abstract

**Background:**

Dysfunction of the glymphatic system (GS), a recently discovered brain by‐product elimination system, is considered to be one of the pathophysiological mechanisms for common neurodegenerative diseases such as Alzheimer's disease (AD), dementia with Lewy bodies (DLB) and Parkinson's disease (PD). In 2017 a new way to assess the GS was proposed – a diffusion tensor images analysis along perivascular spaces (DTI‐ALPS).

In our work we evaluated the DTI‐ALPS index in groups of patients with AD, DLB, PD and in a comparison group of patients with normal pressure hydrocephalus (NPH).

**Methods:**

We examined 32 patients diagnosed with AD [69.5±8.0 years], 15 patients with DLB [71.1±8.0 years], 31 patients with PD without dementia [64.0±6.9 years], 11 patients NPH [68.5±9.8 years] and 27 healthy controls [63.2±7.0 years]. In four patient groups cognitive impairment was rated with Montreal Cognitive Assessment scale (MоCA).

The diffusion images were acquired on a SIEMENS Prisma scanner using a 2D EPI diffusion sequence. TE=82 ms, TR=5600 ms. The b‐values were 1000 and 2500 s/mm². The number of diffusion sampling directions were 64 and 64, respectively. The slice thickness was 2 mm.

Post‐processing steps were performed using DSI studio software. Next, we identified ROIs corresponding to projection and associative fibers with further extraction of tensor values in the directions Dxx, Dyy, Dzz, which were entered into a table to calculate the DTI‐ALPS index using formula ALPS‐index=mean(Dxx_proj_+Dxx_associ_)/mean(Dyy_proj_+Dzz_associ_) for further statistical processing.

**Results:**

Patients with AD, DLB and NPH had a significantly lower values of DTI‐ALPS index on both sides compared to healthy volunteers (p<0.001). The differences between PD and healthy controls turned out to be statistically insignificant.

In addition, analysis of all the patients revealed a significant direct correlation between the MoCA score and DTI‐ALPS values on both sides (p_L_=0.002; p_R_=0.035).

**Conclusion:**

The obtained results showed that the glymphatic dysfunction assessed by DTI‐ALPS index could be considered as a possible participant in the pathogenesis of AD and DLB as well as in NPH reflecting cognitive decline. Whereas the glymphatic dysfunction in PD was not confirmed.

